# Exploring the Potential of Iminosugars as Antivirals for Crimean-Congo Haemorrhagic Fever Virus, Using the Surrogate Hazara Virus: Liquid-Chromatography-Based Mapping of Viral N-Glycosylation and In Vitro Antiviral Assays

**DOI:** 10.3390/pathogens12030399

**Published:** 2023-03-01

**Authors:** Beatrice E. Tyrrell, Abhinav Kumar, Bevin Gangadharan, Dominic Alonzi, Juliane Brun, Michelle Hill, Tehmina Bharucha, Andrew Bosworth, Victoria Graham, Stuart Dowall, Joanna L. Miller, Nicole Zitzmann

**Affiliations:** 1 Department of Biochemistry and Kavli Institute for Nanoscience Discovery, University of Oxford, Oxford OX1 3QU, UK; 2 UK Health Security Agency (UKHSA), Porton Down, Salisbury SP4 0JG, UK

**Keywords:** orthonairovirus, nairovirus, tick-borne virus, antivirals, imminosugars, glycosylation

## Abstract

Crimean-Congo haemorrhagic fever virus (CCHFV) is a pathogen of increasing public health concern, being a widely distributed arbovirus and the causative agent of the potentially fatal Crimean-Congo haemorrhagic fever. Hazara virus (HAZV) is a genetically and serologically related virus that has been proposed as a surrogate for antiviral and vaccine testing for CCHFV. Glycosylation analysis of HAZV has been limited; first, we confirmed for the first time the occupation of two N-glycosylation sites in the HAZV glycoprotein. Despite this, there was no apparent antiviral efficacy of a panel of iminosugars against HAZV, as determined by quantification of the total secretion and infectious virus titres produced following infection of SW13 and Vero cells. This lack of efficacy was not due to an inability of deoxynojirimycin (DNJ)-derivative iminosugars to access and inhibit endoplasmic reticulum α-glucosidases, as demonstrated by free oligosaccharide analysis in uninfected and infected SW13 and uninfected Vero cells. Even so, iminosugars may yet have potential as antivirals for CCHFV since the positions and importance of N-linked glycans may differ between the viruses, a hypothesis requiring further evaluation.

## 1. Introduction

Crimean-Congo haemorrhagic fever (CCHF) is a severe and often fatal disease caused by infection with the tick-borne nairovirus Crimean-Congo haemorrhagic fever virus (CCHFV). CCHFV is one of the most widely distributed arboviruses [1]. This, combined with the lack of therapeutics with proven efficacy [2,3,4], renders CCHFV a pathogen of increasing public health concern.

Post-translational N-glycosylation of viral proteins during their production in the host cell endoplasmic reticulum (ER) can be critical for correct folding and downstream function. Iminosugars are a diverse class of hydroxylated carbohydrate mimics, some of which competitively inhibit ER α-glucosidases [5]. These molecules can interfere with host N-linked glycosylation, conferring broad-spectrum antiviral activity in vitro and in vivo [6,7,8,9,10,11,12]. In order to consider the potential applicability of iminosugar treatment for CCHF patients, at least two mechanistic angles should be considered. The first is whether the virus possesses N-linked glycoproteins that might be misfolded upon iminosugar inhibition of ER α-glucosidases. Indeed, the Gn and Gc surface glycoproteins of CCHFV both possess occupied N-glycosylation sites, which are conserved across known CCHFV strains [13,14]. The second consideration is whether the N-glycosylation of Gn is functionally important: indeed, it is required for the transport of both Gn and Gc to the Golgi apparatus for virion assembly [13,15]. Thus, there is a promising case for testing iminosugars as antivirals against CCHFV.

The severity of CCHF poses a considerable risk to laboratory study; therefore, the related nairovirus Hazara virus (HAZV) was used as a surrogate herein. A lack of reports of pathogenic human infection means that this virus can be safely manipulated at Containment Level (CL) 2, rather than the CL4 required for CCHFV [16,17]. HAZV is both genetically [18] and serologically [19,20] closely related to CCHFV. Furthermore, infection with HAZV has been reported to provide cross-protection against CCHFV in mice [17], and intradermal inoculation of A129 mice with HAZV gave similar clinical signs, pathology and mortality to those reported in murine CCHFV infection models [21]. Consequently, HAZV models have been proposed for the development of antivirals and vaccines for CCHFV [16,21]. SW13 cells have been routinely used as an in vitro model of HAZV infection [21].

Despite the isolation of HAZV in 1964 [22,23], it is relatively little-studied. Early analysis of radiolabelled virus preparations suggested that HAZV possessed three envelope-associated glycoproteins at 30, 45 and 84 kDa, although this was determined by radiolabelled glucosamine incorporation [17] and so could be accounted for by O- rather than N-glycosylation. Indeed, CCHFV glycoproteins have predicted O-glycosylation sites most frequently in the mucin-like domain of M, but also in Gn and Gc sequences [24], and computational analysis has determined regions corresponding to the Gn and Gc of CCHFV in the HAZV M segment [25], indicating the presence of at least two potential O-glycoprotein sequences. However, multiple additional glycoprotein fragments produced from the CCHFV M segment have been identified [26]; thus, HAZV might possess further glycoproteins fulfilling either structural or non-structural roles. Recently, visualisation of surface glycoprotein spikes on HAZV virions has been achieved using cryo-EM, although the precise glycoprotein composition and arrangement could not be determined [27]. In any case, the N-glycosylation status of HAZV glycoproteins has not been confirmed.

Overall, we aimed to establish whether iminosugars have antiviral efficacy against HAZV, and thus potential for treating CCHFV. Specifically, the aims were to confirm whether HAZV expresses N-linked glycoproteins with occupied glycosylation sites; to identify whether iminosugars have antiviral efficacy against HAZV infection in SW13 and Vero cells; and to investigate what effects iminosugar treatment of SW13 and Vero cells may have on the N-glycosylation pathway and HAZV infection, using free oligosaccharide (FOS) analysis.

## 2. Methods

### 2.1. Glycosylation Analyses

#### 2.1.1. Computational Glycosylation Analysis and HAZV and CCHFV Sequence Alignment

For computational prediction of glycosylation sites, the NetNGlyc 1.0 (https://services.healthtech.dtu.dk/service.php?NetNGlyc-1, accessed on 27 May 2020; DTU Health Tech, Lyngby, Denmark), NetOGlyc 4.0 (https://services.healthtech.dtu.dk/service.php?NetOGlyc-4.0, accessed on 1 December 2022; DTU Health Tech, Lyngby, Denmark) and GlycoEP (https://bio.tools/glycoep, accessed on 1 June 2016; Indraprastha Institute of Information Technology, New Delhi, India) [28,29] tools were used to analyse the HAZV JC280 strain glycoprotein sequence (UniProt Knowledge Base [UniProtKB] accession number A6XIP3; Release 12 Oct 2022) and the CCHFV glycoprotein sequence (UniProtKB accession number Q8JSZ3; strain Nigeria/IbAr10200/1970). The CCHFV sequence was aligned with the HAZV glycoprotein using the EMBOSS Needle pairwise sequence alignment tool (EMBL-EBI, Hinxton, UK). The pairwise alignment options used were as follows: matrix = BLOSUM62; gap open = 10; gap extend = 0.5; end gap penalty = false; end gap open = 10; end gap extend = 0.5; output format = pair. The aligned sequences were annotated to highlight the chains of CCHFV and the potential N-glycosylation sites (as shown in UniProt for CCHFV and as predicted by NetNGlyc/GlycoEP for HAZV). Needleman–Wunsch scores were determined for the HAZV aligned sequence regions to each of the CCHFV chains using the EMBOSS Needle pairwise sequence alignment tool with the same alignment options. The theoretical molecular weights of the aligned regions were determined using the Compute pI/Mw tool and peptides covering all potential N-glycosylation sites were determined using in silico trypsin digestion with PeptideCutter (ExPASy, Swiss Institute of Bioinformatics, Lausanne, Switzerland).

#### 2.1.2. HAZV Stocks

HAZV stocks (strain JC280, GenBank accession numbers: L-DQ076419.1, M-DQ813514.1 and S-M86624.1) were generated in SW13 cells (European Collection of Authenticated Cell Cultures, Salisbury, UK [ECACC] 87031801) as previously described [30]. For analysis of glycosylation, HAZV was further purified with 20% sucrose cushion gradient centrifugation (67,000× *g*, 4 °C, 150 min).

#### 2.1.3. HAZV Glycosylation Analysis

For glycosylation analysis with gel shift, HAZV and UDP-glucose:glycoprotein glucosyltransferase (UGGT; a gift from Pietro Roversi) were digested according to the manufacturer’s instructions using PNGase F (enzyme from Pietro Roversi and Dominic Alonzi, Department of Biochemistry, University of Oxford, buffers from New England Biolabs, Hitchin, UK) and endoglycosidase Hf (Endo Hf; New England Biolabs, Hitchin, UK). Samples were denatured by heating with dithiothreitol and NuPAGE^®^ LDS Sample Buffer and run on a NuPAGE^®^ Bis-Tris gel (200 V, 125 mA, 35 min) in NuPAGE^®^ MES SDS Running Buffer (All Thermo Fisher Scientific, Paisley, UK). Protein was stained with InstantBlue (Abcam, Cambridge, UK) and the molecular weights of the sample bands were compared to Novex^®^ Sharp Pre-stained Protein Standard (Thermo Fisher Scientific, Paisley, UK).

#### 2.1.4. In-Gel Digestion and Mass Spectrometry (MS)

In-gel digestion with trypsin and liquid chromatography tandem mass spectrometry (LC-MS/MS) were carried out as previously described using a benchtop Q Exactive hybrid quadrupole-Orbitrap mass spectrometer (Thermo Fisher Scientific, Bremen, Germany) [31]. The data acquired using data-dependent acquisition (DDA) were processed using the Mascot search engine (London, UK) and searching against the NCBInr database. Carbamidomethyl (C) was set as a fixed modification and both oxidation (M) and deamidation (N) were set as variable modifications. In addition to searching with trypsin as the protease, searches were also performed with the enzyme parameter set to ‘None’. Parallel reaction monitoring (PRM) was also carried out using the Skyline software version 18 (MacCoss Lab Software, Seattle, USA) and MS settings as previously described [32]. The HAZV peptides that were targeted were R.LADFFIDTNSSQCYDEILVK.K (88, 107) (N97 deamidated, C101 alkylated; 1190.0567++ or 793.7069+++) and K.NDGPGDHITFCNGSVVTK.I (334, 351) (N346 deamidated, C345 alkylated; 959.9336++ or 640.2915+++).

### 2.2. Iminosugars and Other Compounds

#### 2.2.1. Preparation of Iminosugars and Other Antiviral Compounds

The iminosugars tested comprise *N*-8′-(2′′-tetrahydrofuranyl)-octyl-deoxynojirimycin (2THO-DNJ, UV-12; a gift from Emergent BioSolutions, Gaithersburg, MD, USA, solubilised in acidified water), *N*-butyl-deoxynojirimycin (*N*B-DNJ, Miglustat; a gift from Oxford GlycoSciences Ltd., Abingdon, UK, solubilised in phosphate buffered saline [PBS; Thermo Fisher Scientific, Paisley, UK] or water), *N-*butyl-deoxygalactonojirimycin (*N*B-DGJ; Toronto Research Chemicals, North York, Canada, solubilised in water), *N*-nonyl-deoxynojirimycin (*N*N-DNJ; a gift from Oxford GlycoSciences Ltd., Abingdon, UK, solubilised in dimethyl sulfoxide [DMSO] or acidified water) and *N*N-deoxygalactonojirimycin (*N*N-DGJ; Toronto Research Chemicals, North York, Canada, solubilised in DMSO or acidified water). The non-iminosugar compound ribavirin (Abcam, Cambridge, UK) was solubilised in water. Compounds were assayed for pyrogen contamination and verified to contain less than 0.02 endotoxin units/mL.

#### 2.2.2. Endotoxin Detection Assay

Compounds were subjected to a recombinant Factor-C-based endotoxin test using the EndoZyme^®^ II assay (bioMérieux, Basingstoke, UK) as per the manufacturer’s instructions. Briefly, samples were diluted in endotoxin-free water, added to a 96-well plate in duplicate alongside a standard curve prepared from lipopolysaccharide derived from *Escherichia coli* O55:B5 and warmed to 37 °C. Recombinant enzyme, assay buffer and fluorogenic substrate were added at a ratio of 1:1 (*v:v*) to the sample and the fluorescence was measured for absorption at 380 nm and emission at 445 nm at time 0 and following 1 h of incubation at 37 °C in the dark. Endotoxin concentrations in samples were interpolated from the standard curve produced by fitting a linear regression model to the log10 values of the difference in relative fluorescence. Spike controls were included for each sample and spike recovery was determined to validate the results.

#### 2.2.3. Cytotoxicity Assays

The cytotoxicity of the compounds was assessed with a cell proliferation assay for mitochondrial metabolic activity using a CellTiter 96 AQueous One Solution Cell Proliferation Assay (Promega, Southampton, UK), as per the manufacturer’s instructions. Briefly, cells were cultured with iminosugar in 200 μL culture medium in 96-well tissue culture plates for appropriate durations. After the incubation period, 100 μL of culture medium was removed and 20 μL of a solution containing a tetrazolium compound (3-(4,5-dimethyl-2-yl)-5-(3-carboxymethoxyphenyl)-2-(4-sulfophenyl)-2H-tetrazolium; MTS) and electron coupling reagent (phenazine ethosulfate) was added to the cells in the remaining 100 μL. The samples were incubated for approximately 1–2 h (37 °C, 5% CO_2_), and the absorbance at 490 nm was measured on a SpectraMax M5 microplate reader (Molecular Devices, Wokingham, UK).

### 2.3. Antiviral Assays against HAZV

#### 2.3.1. In Vitro Viral Infection and Drug Treatment

For the assessment of antiviral efficacy of iminosugars against HAZV, confluent cell monolayers in 24-well plates were infected with HAZV diluted to a multiplicity of infection (MOI) of 0.01 in Dulbecco’s Modified Eagle’s Medium (DMEM, Thermo Fisher Scientific, Paisley, UK) without supplements for 60 min (37 °C). Upon removal of the virus, fresh growth medium containing titrations of the drug as indicated was added to cells, which were further incubated for 1, 3 or 6 days for SW13 and 3 days for Vero cells (37 °C, 5% CO_2_). For the analysis of the secreted virus, the supernatant was harvested, diluted 1:5 in AVL buffer (Qiagen, Manchester, UK) and stored at 4 °C until downstream RNA isolation. For the analysis of infectious virus secretion, the supernatant was harvested, aliquoted and stored at −80 °C.

#### 2.3.2. RNA Extraction and HAZV S Segment qRT-PCR

RNA extractions from cell culture supernatants were performed in 96-well plate format using a MagNA Pure 96 System (Roche, Welwyn Garden City, UK) according to the manufacturer’s instructions. HAZV RNA was assayed using an Applied Biosystems 7500 FAST real-time PCR system (Thermo Fisher Scientific, Warrington, UK). Hot-start Taq DNA polymerase was used to enable the Taq-mediated release of fluorescently labelled dyes from a HAZV S-specific probe, using an assay developed and optimised at Porton Down in line with the assay for CCHFV [33]. HAZV S forward (CAAGGCAAGCATTGCACAAC) and reverse (GCTTTCTCTCACCCCTTTTAGGA) primers were used at 0.9 µM and probe (FAM/MGB-TGAAGGATGGGTCAAAGA) at 1.25 µM; all were purchased from Custom (Integrated DNA Technologies, Leuven, Belgium). The reaction mixture was prepared according to the manufacturer’s instructions for Superscript III Platinum One-Step Quantitative RT-PCR kit (Thermo Fisher Scientific, Paisley, UK). Thermal cycling was conducted as follows. Synthesis of cDNA was performed for 10 min at 50 °C, followed by a 2 min denaturation step at 95 °C. PCR thermocycling with fluorescence detection was executed for 45 cycles of 95 °C for 10 s followed by 60 °C for 40 s. Samples were compared to a standard curve generated from serial dilutions of an RNA oligonucleotide designed from the sequence of the HAZV S segment (Integrated DNA Technologies, Leuven, Belgium). Genome equivalents (GEs)/reaction were converted to GE/mL using Microsoft Excel and analysed using GraphPad Prism version 7.01 (GraphPad Software, Inc., Boston, MA, USA).

#### 2.3.3. Settling Plaque Assay

To determine infectious HAZV titres in cell culture supernatants, SW13 and Vero cells were cultured for use in a settling plaque assay. A cell suspension containing 2 × 10^5^ cells/mL in DMEM supplemented with 10% heat-inactivated foetal bovine serum (HI-FBS) was prepared, with 500 µL required per well of a 24-well tissue culture plate used in the plaque assay. Serial 1:10 dilutions of the supernatants were performed (undiluted to 10^−5^ dilution) and 100 µL of supernatant was added to a 24-well plate in duplicate. Media-only and virus stock controls were included. A 500 µL volume of the cell suspension was added to each well, and the plates were mixed by rocking. The plates were incubated on a tray with wet tissue at 37 °C for 4 h. During this time, the plaque assay overlay was constituted by diluting one part 2% carboxmethyl-cellulose in one part 2 × Minimum Essential Medium (MEM). The overlay was mixed vigorously and retained in the incubator at 37 °C until required. A 500 µL volume of the overlay was added to each well by slow pipetting down the side of the well to minimise disruption to the cells, and plates were returned to the incubator for 5 days. Cells were fixed by the addition of 1 mL 20% formaldehyde in PBS per well for at least 1 h. The solution was removed by pipetting and the plates washed with water. To stain the plates, 1 mL of 0.2% crystal violet solution was added to each well and left for around 30 min, until the cells were stained. The dye was removed by pipetting and then the plates were washed with water until the dye was removed. The plates were left to air dry before the plaques were counted by eye. The plaque counts were converted to pfu/mL using Microsoft Excel and analysed using GraphPad Prism version 7.01 (GraphPad Software, Inc., Boston, MA, USA).

#### 2.3.4. FOS Assay

SW13 and Vero cells were infected with HAZV at MOI 0.01 or mock-infected (media-only) and incubated for the indicated times. The cells were washed three times with sterile PBS, removed from the tissue culture plastic by mechanical disruption (scraping) and transferred to microcentrifuge tubes for centrifugation (room temperature, 4000× *g*, 5 min). Cells were lysed with three cycles of freeze-thawing (alternating room temperature and −80 °C) in deionised H_2_O and stored at −80 °C for subsequent protein and FOS assays. FOS were detected as previously described by Alonzi et al. [34]. Briefly, cell lysates were subjected to mixed-bed ion exchange and then lyophilised. FOS were labelled with 2-aminobenzoic acid (2-AA) and purified using a DPA-6S column (Sigma-Aldrich, Gillingham, UK). The unconjugated 2-AA was removed by phase splitting with ethyl acetate for samples from SW13 cells or using Speed Amide-2 cartridges (Applied Separation, Allentown, PA, USA) for samples from Vero cells, and the samples were lyophilised and resuspended in water and then purified using a concanavalin A column. Glycans were separated with normal phase-high performance liquid chromatography (NP-HPLC), and the peak area was used to assess the molar quantity in comparison to standards of known identity and quantity using Empower3 (Waters, Wilmslow, UK). The FOS generation was normalised to the protein concentration assessed using the absorbance at 280 nm measured with a NanoDrop 1000 spectrophotometer (Thermo Fisher Scientific, Paisley, UK).

## 3. Results

### 3.1. Prediction of HAZV N- and O-Glycosylation Sites

To understand whether the presence of glycosylation in HAZV proteins previously identified [17] could be a result of O- rather than N-glycosylation, the HAZV JC280 strain glycoprotein sequence (UniProtKB accession number A6XIP3) was analysed using NetOGlyc 4.0 [29,35]. Fourteen sites with an O-glycosylation potential above a threshold of 0.7 were predicted (Supplementary Table S1), although as NetOGlyc is known to predict more than the true number of O-glycan sites [29], there would be fewer in reality. The four highest scores were for T33, S29, T28 and T34, all of which appear at the N-terminal end of the envelopment polyprotein.

Given that the best-understood antiviral mechanism of action of iminosugars involves the disruption of viral N-linked glycoprotein folding, the presence of N-glycosylation in HAZV proteins was then analysed. According to the UniProtKB entry for CCHFV (Q8JSZ3), the envelopment polyprotein has ten potential N-glycosylation sites (five on the mucin-like variable region, two on GP38, one on Gn and two on Gc). The N-glycosylation sites on Gc and Gn have been confirmed [12]. Through an analysis of further unreviewed CCHFV envelope polyprotein sequences in UniProtKB and in the National Center for Biotechnology Information (NCBI) protein database, we found that N-glycosylation was conserved across all reported CCHFV strains/isolates for GP38 and Gn and was also highly conserved in Gc (>98% of 223 sequences in NCBI). N-glycosylation on the mucin-like variable region was less well-conserved across CCHFV strains/isolates (data not shown).

In contrast, the analysis of the HAZV JC280 strain glycoprotein sequence (A6XIP3) identified eight potential N-glycosylation sites, three of which were predicted to be N-glycosylated, at N97 on GP38, N346 on Gn and N1081 on Gc, after the exclusion of three sequons containing proline (Asn–Pro–Ser/Thr) (Supplementary Figure S1). The remaining two sequons were slightly below the N-glycosylation potential threshold of 0.5 (at N639 on non-structural protein M and N1299 on Gc) (Supplementary Figure S1). The analysis with GlycoEP [28] predicted N-glycosylation at the same five sites (N97, N346, N639, N1081 and N1299) without Asn–Pro–Ser/Thr sequons (Supplementary Table S2). The two sequons below the threshold (without proline) in the NetNGlyc analysis were included in the sequence alignment between CCHFV and HAZV glycoproteins, since these were very close to the threshold, and to see whether CCHFV has potential N-glycosylation sites that align with these sequons in HAZV.

### 3.2. HAZV and CCHFV Sequence Alignment

The EMBOSS Needle pairwise sequence alignment tool was used to align the CCHFV and HAZV sequences (Supplementary Figure S2). The mucin-like variable region of CCHFV with five N-glycosylation sites showed poor alignment with the HAZV sequence, which is over 200 amino acids shorter at the N-terminal end (where the first four potential N-glycosylation sites of CCHFV are present). The HAZV envelopment polyprotein is thought to be processed into four glycoproteins (UniProt A6XIP3 GP_HAZVJ). The GP38 of CCHFV (31 kDa) showed weak alignment to GP38 of HAZV (32 kDa) with a Needleman–Wunsch score of 224.0. The two potential N-glycosylation sites of GP38 in CCHFV at N376 and N426 did not align with any potential N-glycosylation sites of HAZV. The predicted N-glycosylation site of HAZV at N97 also did not align with any potential N-glycosylation sites of CCHFV, indicating that the N-glycosylation of GP38 is very different between these viruses. Gn of CCHFV (32 kDa) showed moderate alignment with Gn of HAZV (32 kDa) with a Needleman–Wunsch score or 642.0. The potential N-glycosylation of CCHFV at N557 aligned well with the predicted N-glycosylation of HAZV at N346. Non-structural protein M of CCHFV (17 kDa) poorly aligned with non-structural protein M of HAZV (13 kDa), with a Needleman–Wunsch score of 22.0. Non-structural protein M of HAZV had a potential N-glycosylation site at N639, although it was below the NetNGlyc 0.5 threshold and did not align with any N-glycosylation site in CCHFV. Gc of CCHFV (72 kDa) showed excellent alignment and similarity with Gc of HAZV (71 kDa), with a Needleman–Wunsch score of 1835.5. The predicted N-glycosylation of HAZV Gc at N1081 aligned with the potential N-glycosylation site for CCHFV Gc at N1345, although this site in CCHFV is not assigned to be glycosylated in the UniProtKB record. In silico digestion of HAZV Gc showed that the tryptic peptide covering N1081 was 88 amino acids in length and therefore not possible to be observed using MS. The potential N-glycosylation site at N1299 of HAZV Gc aligned with the site at N1563 in CCHF Gc. This N1299 site in HAZV Gc was below the NetNGlyc 0.5 threshold, and in silico digestion showed that the tryptic peptide covering this site was 28 amino acids in length and therefore challenging to be detected using MS.

In terms of the O-glycosylation predicted for HAZV, the four residues with the highest scores (T33, S29, T28, and T34) appearing at the N-terminal end of the envelopment polyprotein are likely to be contained within a mucin-like variable region, based on the sequence alignment with CCHFV (Supplementary Figure S2) and the mucin-like O-glycosylated region shown previously [25]. Since the annotated UniProt entry suggests that the HAZV glycoprotein could start with GP38, unlike for CCHFV where the mucin-like variable region comes first, the relatively large number of potential O-glycosylation sites predicted at the HAZV envelopment protein N terminus fits with the mucin-like variable region in CCHFV being most heavily O-glycosylated [24], given that CCHFV GP38 does not have any predicted O-glycans.

### 3.3. HAZV Possesses N-Glycosylated Glycoprotein(s)

**Figure 1:**
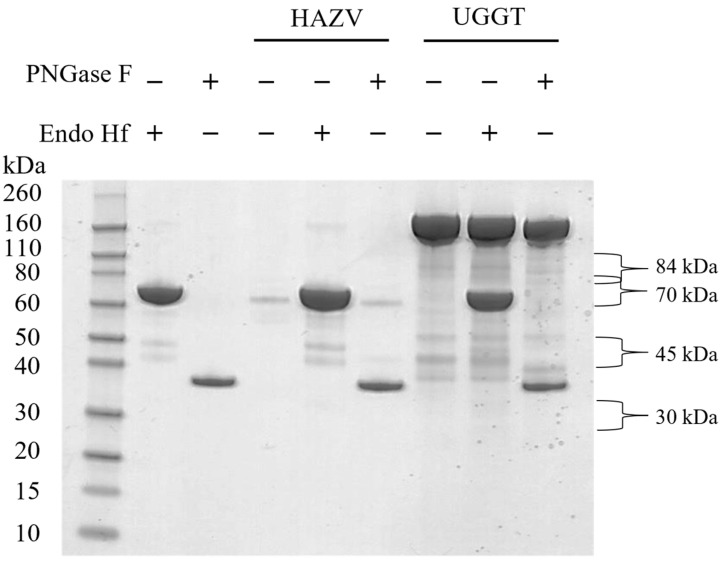
PNGase F- and Endo Hf-digested HAZV and UGGT (a known N-glycosylated protein) run on a 4–12% Bis-Tris gel alongside untreated samples and enzymes.

To investigate whether the predicted N-glycosylation was borne out experimentally, sucrose-cushion-purified HAZV was digested with PNGase F, an endoglycosidase that cleaves the bond between the asparagine residue and the GlcNAc of N-linked oligosaccharides, or Endo Hf, which cleaves the bond between the two GlcNAc residues of the chitobiose core of high mannose and certain complex N-linked oligosaccharides. PNGase F- and Endo Hf-treated HAZV samples were run on a gel alongside HAZV that was not enzymatically treated. UGGT, a known N-glycosylated protein [36], was included as a positive control, for which a gel shift was seen with PNGase F treatment (Figure 1). Strong gel bands at approximately 36 and 70 kDa corresponded to the predicted molecular weights of PNGase F and Endo Hf, respectively. The band corresponding to Endo Hf obscured the major band seen in the HAZV sample; thus, gel shift could not be assessed for this treatment. An apparent gel shift was observed for the major HAZV band at 70 kDa in the untreated vs. PNGase F-treated lanes, suggestive of N-glycosylation; however, the gel bands were sequenced with liquid-chromatography mass-spectrometry (LC-MS) and were identified as mainly bovine serum albumin, with some HAZV nucleocapsid. The levels of the HAZV glycoproteins in the sample were low and there were no bands visible at 30, 45 and 84 kDa. Although bands were not visible, gel pieces were cut at these three molecular weights (since glycoproteins were previously reported at these molecular weights [17]) and proteins in the gel were digested for analysis with LC-MS. Peptides spanning HAZV glycoproteins Gn, GP38 and Gc were identified at 30, 45 and 84 kDa, respectively. The 30 kDa band also had peptides for the HAZV nucleocapsid with a lower score. The HAZV glycoprotein peptides identified are shown in bold in Supplementary Figure S2.

For further analysis, purified HAZV was left untreated or digested with PNGase F in solution, followed by in-solution trypsin digestion and sample analysis by PRM-targeted MS. The detection of a peptide containing an aspartic acid where an asparagine residue appears in the sequence is indicative of an occupied N-glycosylation site, when detected in PNGase F-treated samples but not untreated samples. This analysis identified two occupied N-glycosylation sites at the N97 (peptide sequence LADFFIDTNSSQCYDEILVK) and N346 (peptide sequence NDGPGDHITFCNGSVVTK) residues, while three other tryptic peptides containing potential N-glycosylation sites at N639, N1081 and N1299 were not detected in either treatment condition since they were too long to be detected with MS (the sequence lengths were 54, 88 and 28 amino acids, respectively). The MS/MS spectrum of peptide NDGPGDHITFCNGSVVTK observed with PRM is shown in Supplementary Figure S3. Taken together, these data confirm that HAZV expresses N-linked glycoproteins and therefore was potentially susceptible to iminosugar-mediated ER α-glucosidase inhibition. It was not possible to identify the glycans on HAZV since there was a low amount of protein available and the sample contained highly abundant bovine proteins from the growth medium.

Although the digestion was carried out with trypsin, the Mascot protein search was carried out with the enzyme parameter as ‘None’ to identify any non-tryptic peptides at the N- or C-terminal ends of the glycoprotein chains. The peptide 777FFKGLNSAASK787 was identified (Supplementary Figure S2), which is not an expected tryptic peptide since the amino acid preceding this peptide is a leucine. The alignment data, along with the data for this peptide, confirm that this is HAZV Gc, which starts at F777. Furthermore, the sequence preceding this peptide is 773RKLL776 of HAZV Gc. This RKLL tetrapeptide represents the major cleavage-recognition site present in the glycoprotein precursor of arenaviruses such as Pichinde virus [24]. In CCHFV, the tetrapeptide RRLL is immediately upstream of the cleavage site at the start of Gn, and this tetrapeptide is a cleavage-recognition site in other arenaviruses such as Lassa fever virus [24]. Both CCHFV and HAZV contain this RRLL tetrapeptide (at 516RRLL519 and 304RRLL307, respectively) at the end of GP38.

### 3.4. Iminosugars Lack Antiviral Efficacy against HAZV

**Figure 2:**
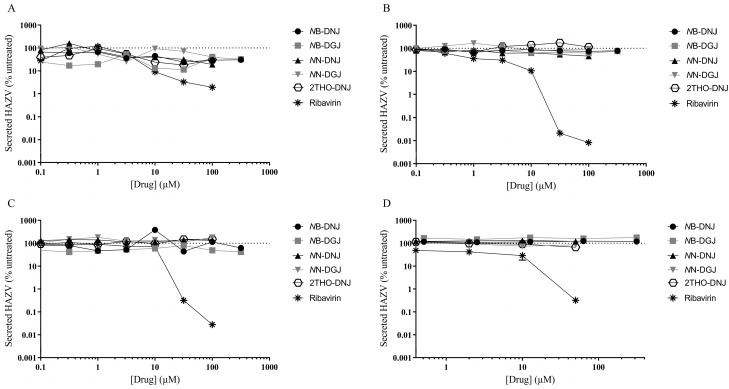
Iminosugars do not have any significant antiviral effect on HAZV secretion in SW13 cells (**A**–**C**) and Vero cells (**D**). SW13 (in technical triplicate) and Vero (single technical replicate) cells were infected with HAZV and treated with the drug and for (**A**) 1, (**B**) 3 or (**C**) 6 days for SW13 cells and (**D**) 3 days for Vero cells. Cell supernatants were collected and HAZV genome equivalents quantified with HAZV S (nucleoprotein)-segment qRT-PCR. HAZV genome equivalents quantified in supernatants from drug-treated cells were normalised to those from untreated cells (set at 100%), and data are presented as mean ± standard deviation.

**Figure 3:**
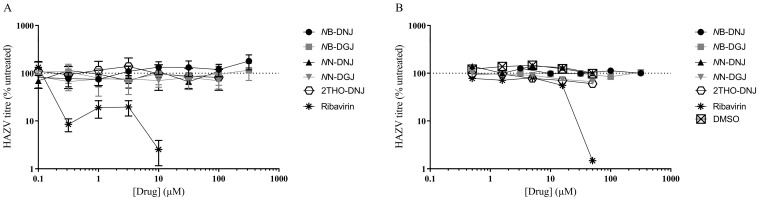
Iminosugars do not reduce infectious HAZV secretion from SW13 cells (**A**) and Vero cells (**B**). SW13 (in technical triplicate) and Vero (single technical replicate) cells were infected with HAZV and treated with the drug as indicated. Cell supernatants were collected after 3 days and HAZV titres quantified with a plaque assay. Plaque counts for supernatants from drug-treated cells were normalised to those from untreated cells (set at 100%), and data are presented as mean ± standard deviation.

Following established methods for culturing HAZV, the SW13 human adrenal adenocarcinoma and Vero African green monkey kidney epithelial cell lines were utilised for in vitro antiviral efficacy testing. Ribavirin was included as a putative positive control, since this compound was previously shown to inhibit the release of infectious HAZV from A549 human lung adenocarcinoma cells [16]. A range of iminosugars was tested against HAZV at non-toxic concentrations (Supplementary Figure S4). The supernatant was collected from cells at 1, 3 and 6 days post-infection to measure the total virus secretion with qRT-PCR detecting S (nucleoprotein)-segment RNA. Since iminosugars might impact the infectivity of virions rather than their secretion, infectious virus released to the supernatant was quantified with a plaque assay for Day 3 supernatant samples. By both measures, ribavirin demonstrated potent antiviral efficacy, whereas neither 2THO-DNJ, *N*N-DNJ or *N*N-DGJ (at concentrations of up to 100 μM) nor *N*B-DNJ or *N*B-DGJ (at concentrations of up to 316 μM) possessed notable antiviral efficacy in terms of reducing the total HAZV secretion (Figure 2) or the release of infectious HAZV (Figure 3), suggesting that iminosugars do not have antiviral potential against HAZV.

### 3.5. FOS Assay Confirms That SW13 and Vero Cells Are Susceptible to Iminosugar Treatment

Since iminosugars showed a lack of antiviral efficacy against HAZV, confirmation was sought that this was not due to a lack of susceptibility of the SW13 and Vero cells used to iminosugar treatment. Therefore, both uninfected and HAZV-infected SW13 and uninfected Vero cells were treated with iminosugars under conditions reflecting the antiviral assay. Subsequently, the cell lysates were subjected to a FOS assay, involving the purification and HPLC-mediated identification of diagnostic oligosaccharide species generated upon the inhibition of ER α-glucosidases, namely Glc_3_Man_7_GlcNAc_2_ and Glc_1_Man_4_GlcNAc_1_, indicative of ER α-glucosidase I and II inhibition, respectively. Detection of FOS is dependent on the activity of the N-linked glycosylation pathway, such that there is a flux of ER α-glucosidase substrates that will lead to FOS generation upon enzyme inhibition, and on the accessibility of the glucosidases to iminosugars. This may vary between cell types due to variable membrane compositions imposing different permeability barriers to iminosugar entry [5,37], as well as potential roles of transporters in iminosugar uptake [38].

**Figure 4:**
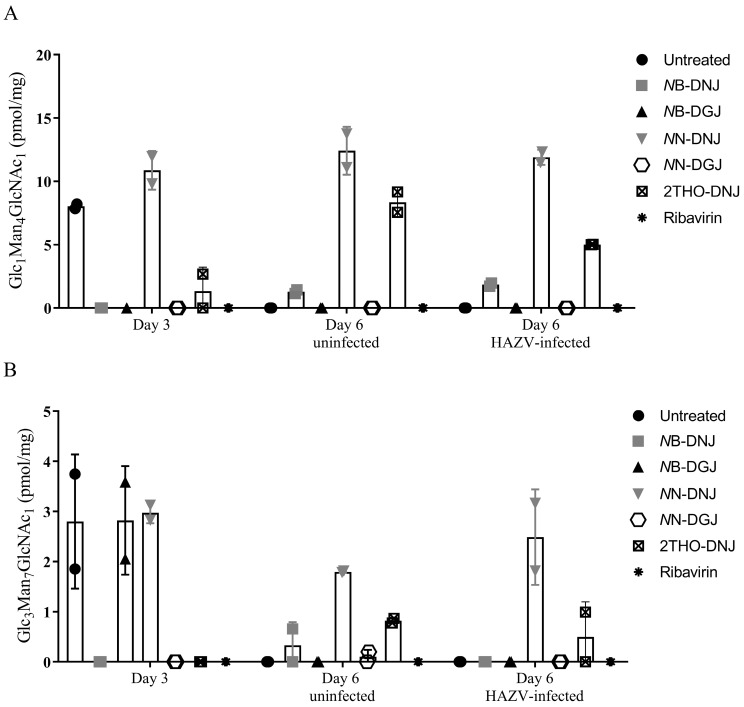
HAZV infection reduces some iminosugar-induced FOS accumulation in SW13 cells. SW13 cells (assayed in technical duplicate) were left untreated or treated with 316 μM *N*B-DNJ or *N*B-DGJ or 100 μM 2THO-DNJ, *N*N-DNJ, *N*N-DGJ or ribavirin or for 3 days (Day 3 samples) prior to mock or HAZV infection, after which the same drug treatment was continued for 3 days (Day 6 Uninfected or Day 6 HAZV-infected samples). Cells were lysed and FOS species purified and detected with NP-HPLC. The Glc_1_Man_4_GlcNAc_1_ (**A**) and Glc_3_Man_7_GlcNAc_2_ (**B**) FOS species detected, diagnostic for ER α-glucosidase II and I inhibition, respectively, were normalised to total protein content and plotted as mean ± standard deviation. Due to experimental failure/loss of sample, there is only one experimental data point for *N*B-DGJ in (**A**).

The analysis of uninfected and HAZV-infected SW13 cells identified an accumulation of FOS species, diagnostic for inhibition of both ER α-glucosidases upon treatment with DNJ-derivative iminosugars (Supplementary Figure S4). Similar results were seen in uninfected Vero cells (Supplementary Figure S5). As expected, ER α-glucosidase inhibition was not seen for treatment with ribavirin or DGJ-derivative iminosugars in both cell types, although there was some background for the Glc_3_Man_7_GlcNAc_2_ detected, particularly in the samples taken after 3 days for SW13 cells (Figure 4 and Supplementary Figure S6). Detection of these FOS species in DNJ-derivative iminosugar-treated cells indicated that the ER α-glucosidases in SW13 and Vero cells were accessible and susceptible to inhibition by iminosugars.

Interestingly, the ER α-glucosidase II inhibition mediated by some iminosugar treatments appeared to be reduced in the context of HAZV infection compared to uninfected cells (Supplementary Figure S4). This was unexpected, since increased glycoprotein turnover upon infection with an N-linked glycoprotein-expressing virus, such as HAZV, might be expected to result in increased FOS generation on iminosugar treatment. To investigate the reason for this, SW13 cells were pre-treated with iminosugar for 3 days, prior to infection with HAZV or mock-infection, and treatment continuation for a further 3 days. Cell lysates were collected and FOS assays performed. Diagnostic FOS species for ER α-glucosidase I and II inhibition were observed with the DNJ-derivative treatment, as expected, although a surprising peak indicative of ER α-glucosidase I inhibition was observed at Day 3 (Figure 4), which was not consistent with previous analyses. A reduction in the Glc_1_Man_4_GlcNAc_1_ FOS species accumulated in response to 2THO-DNJ-mediated ER α-glucosidase II inhibition was again observed (Figure 4 as in Supplementary Figure S4C). This suggests that the accumulation of FOS indicative of iminosugar-mediated ER α-glucosidase II inhibition may be reduced in the context of HAZV infection.

## 4. Discussion

CCHF represents a disease of increasing public health concern, with no vaccine and no antivirals established to be effective against CCHFV [2,39,40]. While ribavirin efficacy has been suggested by some, but not all, preclinical data [41,42,43,44,45,46], its clinical use is controversial given the lack of supporting evidence [4,40,47]; therefore, further antiviral drug development is required. The conserved N-glycosylation of the structural Gn and Gc glycoproteins of CCHFV, known to be of functional importance for virion assembly [13,14,15], renders CCHFV a promising target for DNJ-derivative iminosugars that inhibit ER α-glucosidases. In line with this, viral glycoprotein misfolding has demonstrated antiviral efficacy against diverse viruses [8]. However, the severity of CCHF means that the genetically and serologically related HAZV has been proposed as a surrogate for testing antivirals and vaccines against CCHFV [16,21]. Antiviral activity of iminosugars has been demonstrated for other viruses in Vero cells [48]; thus, HAZV infection of SW13 and Vero cells was used for iminosugar antiviral efficacy testing in this study as a putative surrogate for CCHFV.

Initially, the presence of N-glycosylation was confirmed at two of the putative sites in the HAZV glycoprotein sequence, confirming the potential suitability of iminosugar-induced N-glycoprotein misfolding as an antiviral strategy for HAZV. However, the apparent differences between the N-glycosylation sites of HAZV and CCHFV glycoproteins indicated that the functional significance of N-glycosylation could differ between the viruses. The antiviral efficacy of a panel of iminosugars against HAZV was then investigated. Ribavirin was effective as a positive control [16], whereas the tested iminosugars lacked any substantial antiviral effect on HAZV virion secretion or infectious virus titres in either of the two cell culture models tested. In order to confirm this was not because of a lack of susceptibility of the cells to iminosugar treatment, a FOS assay was conducted for uninfected and HAZV-infected cells treated with iminosugars, finding that ER α-glucosidase inhibition was evident under both conditions. Thus, the lack of antiviral effect was not accounted for by enzyme inaccessibility. However, the amount of FOS accumulation seen in uninfected SW13 appeared to be considerably lower than that generated upon iminosugar treatment of primary human cell types [49]. Cell-type-dependent iminosugar efficacy has previously been described for influenza virus [5] and dengue virus [48,50,51] and may be related to variation in membrane composition determining access to the ER [37], the presence of putative iminosugar transporters [38] or differences in glycoprotein-processing pathways between cell types [52,53].

An alternative explanation for the lack of antiviral effect of iminosugars against HAZV could be that while a single N-linked glycan can be sufficient to endow susceptibility to iminosugar treatment [54], N-glycosylation of a glycoprotein does not necessitate reliance on the calnexin cycle, entry to which is controlled by ER α-glucosidase II. Alternative chaperones such as BiP may aid the folding of N-glycosylated viral glycoproteins [55], or N-glycosylation may be dispensable for the folding or function of a glycoprotein. Thus, virion formation and infectivity may be preserved regardless of iminosugar treatment of a virus possessing N-linked glycoproteins. This is not without precedent: strain-specific iminosugar efficacy is seen for the influenza and vesicular stomatitis viruses [5,56].

The suitability of HAZV as a surrogate for the antiviral efficacy testing of iminosugars against CCHFV is called into question by the differences in N-glycosylation of their viral glycoproteins. Therefore, iminosugar efficacy might still be assessed directly against CCHFV infection, although the requirement for CL4 facilities [21] and experimental challenges in the accurate quantification of infectious virus remain barriers to this line of enquiry. Although the CCHFV minigenome or viral-like particle (VLP) systems [57] are useful tools for testing therapeutics targeting aspects such as viral replication, they are unsuitable for the assessment of iminosugar efficacy as they lack the viral glycoprotein-encoding M segment. Future development of a reverse genetics system for HAZV could provide a valuable resource for iminosugar efficacy testing against a recombinant virus possessing CCHFV glycoproteins, if the CCHFV M segment was incorporated into VLPs with a HAZV background. Using this system, the infectivity of VLPs resulting from iminosugar-treated cells could be determined as a measure of iminosugar efficacy. Such a system would also enable the comparison of antiviral efficacy between HAZV and CCHFV glycoprotein-containing VLPs, identifying whether any differences in iminosugar efficacy could be attributed to the respective viral glycoproteins.

In summary, the antiviral efficacy of iminosugars against HAZV was explored, as a proposed surrogate virus for CCHFV antiviral drug development. The occupation of two N-glycosylation sites in the HAZV glycoprotein was confirmed, supporting the possibility for iminosugar-mediated glycoprotein misfolding as a mechanism of action against the virus. However, no antiviral efficacy was observed following iminosugar treatment of HAZV-infected SW13 and Vero cells, despite ER α-glucosidase inhibition being achieved. Since the glycosylation of CCHFV and HAZV glycoproteins appears to differ, and for CCHFV the N-glycosylation of its glycoproteins is of functional importance, iminosugars may yet hold potential as antivirals against CCHFV. However, further pursuit of this hypothesis would likely require direct evaluation of iminosugar antiviral efficacy against CCHFV.

## Data Availability

The data presented in this study are available in the article and its accompanying Supplementary Material.

## Supplementary Materials

The following supporting information can be downloaded at: https://www.mdpi.com/article/10.3390/pathogens12030399/s1, Table S1: O-glycosylation prediction of HAZV and CCHFV; Table S2: Predicted N-glycosylation of HAZV glycoprotein using GlycoEP; Figure S1: N-glycosylation prediction of HAZV; Figure S2: Sequence alignment of CCHFV with HAZV glycoprotein; Figure S3: MS/MS spectrum of peptide NDGPGDHITFCNGSVVTK; Figure S4: Uninfected and HAZV-infected SW13 cells are susceptible to iminosugar-mediated ER α-glucosidase inhibition; Figure S5: Cytotoxicity of iminosugars and ribavirin in SW13 and Vero cells; Figure S6: Uninfected Vero cells are susceptible to iminosugar-mediated ER α-glucosidase inhibition.
