# Supplementary material for: Exploring the Potential of Iminosugars as Antivirals for Crimean-Congo Haemorrhagic Fever Virus, Using the Surrogate Hazara Virus: Liquid-Chromatography-Based Mapping of Viral N-Glycosylation and In Vitro Antiviral Assays
Source: Pathogens. 2023 Mar 1;12(3):399. doi: 10.3390/pathogens12030399 (PMC10057787; doi:10.3390/pathogens12030399)
Supplement: Supplementary file 1 [file pathogens-12-00399-s001.zip › pathogens-2160261-supplementary.pdf]

Supplementary Tables and Figures for:

Exploring the Potential of Iminosugars as Antivirals for Crimean-Congo Haemorrhagic Fever Virus, Using the Surrogate Hazara Virus: Liquid-Chromatography-Based Mapping of Viral N-Glycosylation and In Vitro Antiviral Assays

Beatrice E. Tyrrell, Abhinav Kumar, Bevin Gangadharan, Dominic Alonzi, Juliane Brun, Michelle Hill, Tehmina Bharucha, Andrew Bosworth, Victoria Graham, Stuart Dowall, Joanna L. Miller and Nicole Zitzmann

Correspondence to: nicole.zitzmann@bioch.ox.ac.uk

Supplementary Table S1. O-glycosylation prediction of HAZV and CCHFV. Scores from the NetOGlyc 4.0 Server (DTU Bioinformatics, University of Denmark). A total of 14 sites on HAZV and 90 sites on CCHFV had O-glycosylation potentials above a threshold of 0.7. The gradation of colours relate to the O-glycosylation score where the highest scores are shown in darker green, lowest scores are in darker red and scores in between are in yellow. In both viruses, the O-glycosylation potential is highest in the mucin-like variable region.

Chain	HAZV		CCHFV	
	Position	Score	Position	Score
Mucin-like variable region	22	0.827772	23	0.931262
	24	0.829553	27	0.972228
	28	0.959723	32	0.966068
	29	0.969206	34	0.952545
	33	0.975653	36	0.965209
	34	0.918613	37	0.988626
			43	0.977169
			44	0.980107
			49	0.965769
			50	0.939262
			53	0.875142
			55	0.968651
			59	0.992849
			60	0.978914
			62	0.987548
			64	0.986252
			65	0.994089
			68	0.93942
			73	0.905363
			78	0.959212
			79	0.974422
			83	0.971223
			84	0.956699
			86	0.927088
			90	0.982535
			92	0.974401
			93	0.978733
			99	0.989353
			100	0.977282
			102	0.903175
			104	0.939893
			106	0.973813
			113	0.98995
			114	0.943063
			116	0.988119
			120	0.988506
			121	0.993504

			124	0.996035
			127	0.97703
			128	0.981646
			132	0.993158
			134	0.99748
			135	0.981392
			137	0.99253
			138	0.985115
			142	0.973401
			151	0.932734
			153	0.976887
			154	0.990018
			160	0.975784
			161	0.96955
			162	0.977372
			164	0.976607
			166	0.924413
			170	0.987979
			171	0.963666
			173	0.970329
			174	0.978832
			175	0.982323
			180	0.995965
			185	0.993082
			189	0.972056
			194	0.981983
			198	0.939476
			202	0.979987
			207	0.942452
			209	0.930992
			211	0.987156
			213	0.9466
			218	0.913611
			219	0.977484
			221	0.9736
			228	0.990185
			230	0.982063
			233	0.939943
			237	0.970974
			240	0.973973
			242	0.982473
GP38	113	0.738189	323	0.771324
			326	0.933441
			329	0.938859
			330	0.949821
			332	0.983169

			333	0.94856
			339	0.759312
			378	0.726875
			381	0.936315
			382	0.876265
			387	0.823809
Non-Structural protein M	633	0.856664		
	638	0.76701		
	679	0.903456		
	683	0.911063		
	685	0.828454		
	698	0.756311		
Gc	894	0.875082	1399	0.771154

CCHFV, Crimean-Congo haemorrhagic fever virus; HAZV, Hazara virus.

Supplementary Table S2. Sites predicted to be N-glycosylated in the HAZV glycoprotein (UniProtKB accession number A6XIP3) using GlycoEP (N-linked glycosylation prediction based on binary profile of patterns using default SVM 0.0 thresholds. The darker the green colour for the score cells, the higher the score. N41 and N159 were excluded due to the prolines (P) in the sequons and since the scores were very low.

Position	Sequon	Score
41	NPS	0.189
97	NSS	1.069
159	NPS	0.33
346	NGS	0.637
639	NQS	1.23
1081	NST	0.919
1299	NET	0.801

HAZV, Hazara virus.

Supplementary Figure S1. N-glycosylation prediction of HAZV. Modified output from the NetNGlyc 1.0 Server (DTU Bioinformatics, University of Denmark). Default settings were used where predictions were done only on the Asn-Xaa-Ser/Thr sequons. All Asn-Pro-Ser/Thr sequons were excluded and the remaining sequons were included for sequence alignment. A total of eight Asn-Xaa-Ser/Thr sequons were identified (highlighted in blue). Three of these were Asn-Pro-Ser/Thr sequons (Asn shown in red at N41, N159 and N930) which were excluded. Two sequons (Asn shown in orange at N639 and N1299) were below the N-glycosylation potential threshold of 0.5 (at at 0.4717 and 0.4993 respectively) with long tryptic peptides and therefore were not considered for analysis by mass spectrometry but were included in the sequence alignment to see if CCHFV has potential N-glycosylation sites which align with these sequons of HAZV. The remaining three sequons (Asn shown in green at N97, N346 and N1081) were considered to have potential N-glycosylation sites and were selected for further analysis by mass spectrometry.

```
Asn-Xaa-Ser/Thr sequons in the sequence output below are highlighted in blue.

          Asparagines predicted to be N-glycosylated are highlighted in green.
```

Output for HAZV glycoprotein sequence (Uniprot accession number A6XIP3)

```
Name:  HAZV glycoprotein       Length:  1421
MEGSYWWLSLLALLAWGANGESTSPAETSPAPTTPNPPVVNPSLRRKIVNQRILSAMGMDSDPSNEALNGVCQSIHSNGC      80
NANELKLRLADFFIDTNSSQCYDEILVKKPCSSLTPAHNSHWVPRGLDKSEVDKIFDTKLKLFFSQSRKVTCLSASALNP     160
SQFVKHFQVKIQETSGPAKQSLRSLHCVNLVWSHSHKGEKEVVHVLQSAVPVKLKNCLAMLNFRQCYYNQQSEGPVVVPS     240
YQHNGEKWVTGAYTMTVEVDKHADGPCEISTTCITEGSEIKPGVHSLRGFKTTLVIHGKRNTGRRLLSSSNARQECSSGT     320
FLGEGGSAQVVGPKNDGPGDHITFCNGSVVTKIRLGQEHGCYTVRRIKTYRNCRPEEGSSACEVDDELKPCGAQKCMNVH     400
LSVKGLVKTSRGSNVQVHSCDKDCLIQIPEGFGDIQIDCPGGTQHYLESNVLDVDCPMYNRLGGLMLYFCRMSHRPRTCL     480
ALFIWLGAGYGITCIAGYMVYYAILALSMLTRCLKRKYMVKGDFCLKCEQKCVTSLDQTLHDESCSYNICPYCGNRLPEE     560
GLRRHVPSCPKRKQRLEEIDLYLDYLLVPCPLHFALSTAVKLGTLLKRLSWVTVFLCLFLTAIAPVQGQVTTSPVLPSNQ     640
STECTLLPPPVFLIFSAVLMSKTLKRMGPVNKVGAAGHSARRTNSPKNLYKSKQIANTKSGPREPRRRVVVKALLILTAS     720
SALQSIHLAQAFDSGSLPEGAWEEEMQLVQGCNQECSLEEDECSCPDGQSMTRKLLFFKGLNSAASKMASSHRLLTSVSI     800
DTPWGAIKVESTYKPRLASSNIQLAWNSIEEQGDKVILSGKSTSIIKLEEKTGMQWSLGSESAAEEKRLLVSILDYTQVY     880
SSTFQYITGDRTVSEWPKATCTGDCPDRCGCSTSSCLYKSWPHSRNWRCNPTWCWGVGTGCTCCGVDILRPFNKYFVTKW     960
TTEYVRTDVLVCVELTDQERHCDVVEAGSQFVIGPVRVVVSDPQNVQTKLPSEILTIQKLEGNQVVDIMHATSIVSAKNA    1040
CKLQSCTHGSPGDMQILHTDNLIQHSHDGGLNLADLNPLVNSTWMSWEGCDLDYYCTTGSWPSCTYTGINSENTESFDNL    1120
LNTESNLCERFHFHSKRISASGSTLQMDLKGRPNSGGGELSVLVDVKGLELHSKKISLKGLSFKTLSCSGCYACSSGLSC    1200
TVEVRIERPDEFTVHLRSVSPDIAVAEGSIIARRMTGGPLSRLRAFAVRKVKKICFEIVEKSYCKDCKNEDTTKCIEVEL    1280
QPPKDILLEHKGTIIKRQNETCVSGLQCWTESASSFVSGVGSFFRNYLGSITLGIVLTLLPVAVVLLFFCYGDKLFKLCS    1360
CFRCCRGLSRGKVRKELDEDELRNKLKKFSKEGELFGKEKKDARTIALLLSGKGKNYKELV
```

```
................................................................................      80
................N...............................................................     160
................................................................................     240
................................................................................     320
.........................N......................................................     400
................................................................................     480
................................................................................     560
................................................................................     640
................................................................................     720
................................................................................     800
................................................................................     880
................................................................................     960
................................................................................    1040
........................................N.......................................    1120
................................................................................    1200
................................................................................    1280
................................................................................    1360
.............................................................                       1440
```

(Threshold=0.5)

SeqName	Position	Potential	Jury agreement	N-Glyc result	
HAZV	41 NPSL	0.6857	(9/9)	++	WARNING: PRO-X1.
HAZV	97 NSSQ	0.6163	(8/9)	+	
HAZV	159 NPSQ	0.7756	(9/9)	+++	WARNING: PRO-X1.
HAZV	346 NGSV	0.5580	(7/9)	+	
HAZV	639 NQST	0.4717	(7/9)	-	
HAZV	930 NPTW	0.5820	(8/9)	+	WARNING: PRO-X1.
HAZV	1081 NSTW	0.5839	(8/9)	+	
HAZV	1299 NETC	0.4993	(5/9)	-	

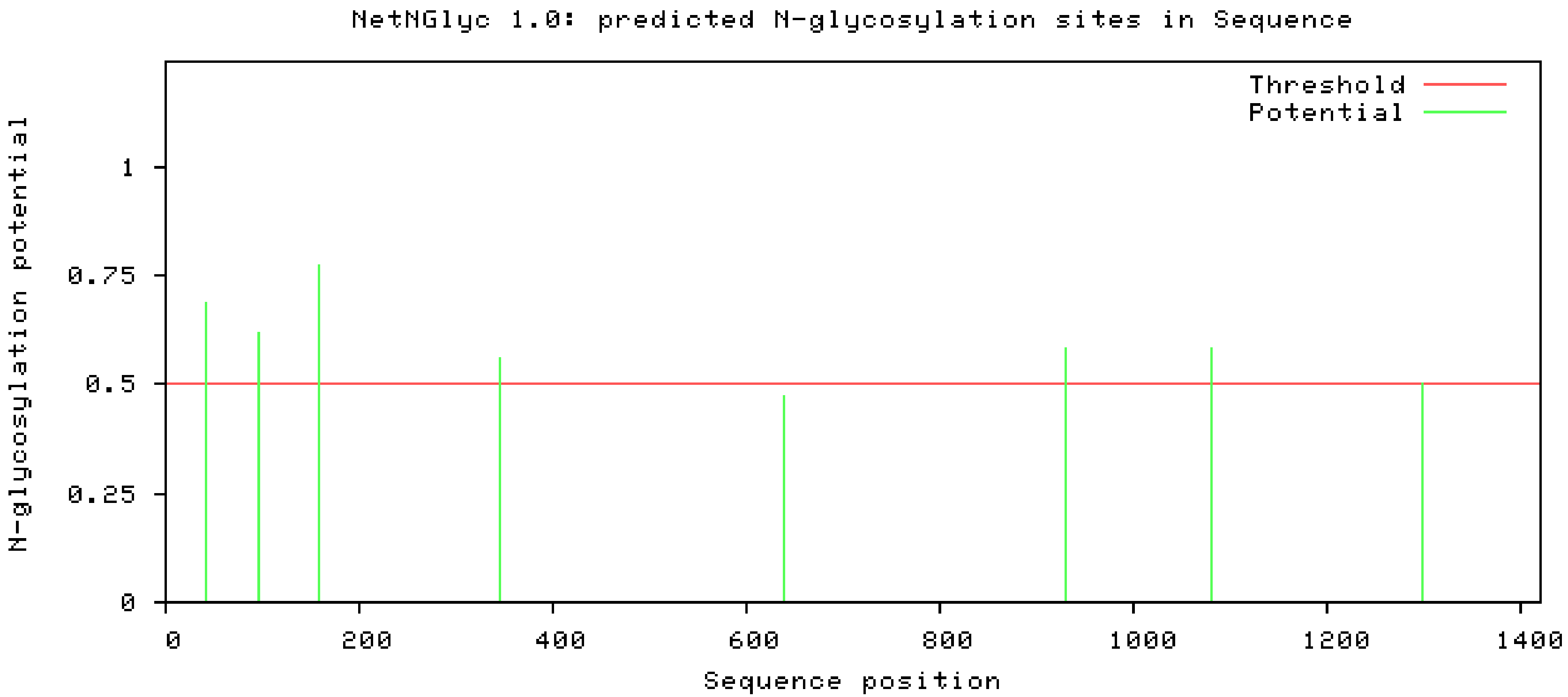

CCHFV, Crimean-Congo haemorrhagic fever virus; HAZV, Hazara virus.

Supplementary Figure S2. Sequence alignment of CCHFV (strain Nigeria / IbAr10200 / 1970; Uniprot Q8JSZ3) with HAZV glycoprotein (Uniprot A6XIP3). Chains are as currently shown on Uniprot (Release 12-Oct-2022). All HAZV glycoprotein peptides identified by mass spectrometry are shown in bold.

Mucin-like variable region

GP38

Glycoprotein N

Non-Structural protein M

Glycoprotein C

Propeptide

N = Potential N-glycosylation sites (as shown in Uniprot for CCHF/HAZV and as predicted by NetNGlyc 1.0 and GlycoEP for HAZV).

Underlined = Tryptic peptides covering N-glycosylation sites.

Bold in blue = Peptides identified in 84 kDa band.

Bold in green = Peptides identified in 45 kDa band.

Bold in yellow = Peptides identified in 30 kDa band.

HAZARA = Hazara virus glycoprotein (Uniprot A6XIP3)

CCHFV = Crimean-Congo hemorrhagic fever virus Envelopment polyprotein strain Nigeria / IbAr10200 / 1970 (Uniprot Q8JSZ3)

```
                                              10        20
CCHFV                                 MHISLMYAILCLQLCGLGETHGSHNE

         30        40        50        60        70        80
CCHFV   TRHNKTDTMTTPGDNPSSEPPVSTALSITLDPSTVTPTTPASGLEGSGEVYTSPPITTGS

         90        100       110       120       130       140
CCHFV   LPLSETTPELPVTTGTDTLSAGDVDPSTQTAGGTSAPTVRTSLPNSPSTPSTPQDTHHPV

        150       160       170       180       190       200
CCHFV   RNLLSVTSPGPDETSTPSGTGKESSATSSPHPVSNRPPTPPATAQGPTENDSHNATEHPE

        210       220       230       240       250       260
CCHFV   SLTQSATPGLMTSPTQIVHPQSATPITVQDTHPSPTNRSKRNLKMEIILTLSQGLKKYYG
                       :                                       :   :
HAZARA           MEGSYWWLSLLALLAWGANGESTSPAETSPAPTTPNPPVVNPSLRRKIVNQ
```

```
                        10        20        30        40        50

        270       280       290       300       310         320
CCHFV  KILRLLQLTLEEDTEGLLEWCKRNLGLDCDDTFFQKRIEEFFITGEGH--FNEVLQFRTP
       .::  . .  . ..:.:   :.   .  :. . .. :. .:::  ..   ..:.:  .
HAZARA RILSAMGMDSDPSNEALNGVCQSIHSNGCNANELKLRLADFFIDTNSSQCYDEILVKKPC
              60        70        80        90       100       110

          330          340       350       360       370       380
CCHFV  GTLSTTEST---PAGLPTAEPFKSYFAKGFLSIDSGYYSAKCYSGTSNSGLQLINITRHS
       ..:. ....   : ::  .:  : . .:  : . :   .. : :... .  :...  . .
HAZARA SSLTPAHNSHWVPRGLDKSEVDKIFDTKLKLFF-SQSRKVTCLSASALNPSQFVK--HFQ
             120       130       140        150       160

             390       400       410       420       430       440
CCHFV  TRIVDTPGPKITNLKTINCINLKASIFKEHREVEINVLLPQVAVNLSNCHVVIKSHVCDY
       ..: .: ::   .:....:.::  :  ..  ..  ..::   : :.:.:: .... . : :
HAZARA VKIQETSGPAKQSLRSLHCVNLVWSHSHKGEKEVVHVLQSAVPVKLKNCLAMLNFRQCYY
      170       180       190       200       210       220

             450        460       470       480       490       500
CCHFV  SLDIDGAVRLPHIYHEGV-FIPGTYKIVIDKKNKLNDRCTLFTDCVIKGREVRKGQSVLR
       . . .: : .:   :.:  .. :.: ....  .. .  : . : :. .: :.. :   ::
HAZARA NQQSEGPVVVPSYQHNGEKWVTGAYTMTVEVDKHADGPCEISTTCITEGSEIKPGVHSLR
      230       240       250       260       270       280

               510        520       530       540       550
CCHFV  QYKTEIRI-GKASTGSRRLL-SEEPSDDCISRTQLLRTETAEIHGDNYGGPGDKITICNGS
        .:: . : :: .:: :::: : .  ..: : : : .  .:.. : .  ::::.::.::::
HAZARA GFKTTLVIHGKRNTG-RRLLSSSNARQECSSGTFLGEGGSAQVVGPKNDGPGDHITFCNGS
      290       300        310       320       330       340

     560       570       580       590       600       610
CCHFV  TIVDQRLGSELGCYTINRVRSFKLCENSATGKNCEIDSVPVKCRQGYCLRITQEGRGHVK
       ...  :::.: ::::. :..... :.    .. ::.:.    :    :. .    .: ::
HAZARA VVTKIRLGQEHGCYTVRRIKTYRNCRPEEGSSACEVDDELKPCGAQKCMNVHLSVKGLVK
      350       360       370       380       390       400

     620       630       640       650       660       670
CCHFV  LSRGSEVVLDACDTSCEIMIPKGTGDILVDCSGGQQHFLKDNLIDLGCPKIPLLGKMAIY
        ::::.: . .:: .: :.::.: ::: .:: :: ::.:..:..:. ::    :: . .:
HAZARA TSRGSNVQVHSCDKDCLIQIPEGFGDIQIDCPGGTQHYLESNVLDVDCPMYNRLGGLMLY
      410       420       430       440       450       460

     680       690       700       710       720       730
CCHFV  ICRMSNHPKTTMAFLFWFSFGYVITCILCKAIFYLLIIVGTLGKRLKQYRELKPQTCTIC
       .::::..:.: .:...:.. :: ::::    ..: .. .. : . ::.   .: . :  :
HAZARA FCRMSHRPRTCLALFIWLGAGYGITCIAGYMVYYAILALSMLTRCLKRKYMVKGDFCLKC
      470       480       490       500       510       520

     740       750       760       770       780       790
CCHFV  ETTPVNAIDAEMHDLNCSYNICPYCASRLTSDGLARHVIQCPKRKEKVEETELYLNLERI
       :   :...:  .:: .:::::::::..::  .:: ::: .::::...::  .:::.   .
HAZARA EQKCVTSLDQTLHDESCSYNICPYCGNRLPEEGLRRHVPSCPKRKQRLEEIDLYLDYLLV
      530       540       550       560       570       580
```

```
        800       810       820       830       840       850
CCHFV     PWVVRKLLQVSESTGVALKRSSWLIVLLVLFTVSLSPVQSAPIGQGKTIEAYRAREGYTS
          :  ..  :... . :. ::: ::. :.: :: ....:::    ::  :  .  . .  ..
HAZARA    PCPLHFALSTAVKLGTLLKRLSWVTVFLCLFLTAIAPVQ----GQVTTSPVLPSNQ--ST
         590       600       610       620           630       640

        860       870       880       890       900       910
CCHFV     ICLFVLGSILFIVSC-LMKGLVDSVGNSFFPGLSICKTCSISSINGFEIESHKCYCSLFC
           : ..   ...: :  ::.  .  .:                 .:     .:.
HAZARA    ECTLLPPPVFLIFSAVLMSKTLKRMG----------------PVNKVGAAGHSA------
               650       660                       670       680

         920       930       940       950       960       970
CCHFV     CPYCRHCSTDKEIHKLHLSICKKRKKGSNVMLAVCKLMCFRATMEVSNRALFIRSIINTT
              :. .. :...:         :. .:.              . . :    : ....
HAZARA    ----RRTNSPKNLYK--------SKQIANT--------------KSGPREPRRRVVVKAL
                     690                               700       710

         980       990       1000      1010      1020      1030
CCHFV     FVLCILILAVCVVSTSAVEMENLPAGTWEREEDLTNFCHQECQVTETECLCPYEALVLRK
          ..:        .  ..: .  .:: :.::.: .:.. :.:::.. : :: ::    . ::
HAZARA    LILTASSALQSIHLAQAFDSGSLPEGAWEEEMQLVQGCNQECSLEEDECSCPDGQSMTRK
             720       730       740       750       760       770

        1040      1050      1060      1070      1080      1090
CCHFV     PLFLDSTAKGMKNLLNSTSLETSLSIEAPWGAINVQSTYKPTVSTANIALSWSSVEHRGN
           ::. .  .. ... .:  : ::.::..:::::.:.::::: ....:: :.:.:.:..:.
HAZARA    LLFFKGLNSAASKMASSHRLLTSVSIDTPWGAIKVESTYKPRLASSNIQLAWNSIEEQGD
             780       790       800       810       820       830

        1100      1110      1120      1130      1140      1150
CCHFV     KILVSGRSESIMKLEERTGISWDLGVEDASESKLLTVSVMDLSQMYSPVFEYLSGDRQVG
          :...::.: ::.::::.::..:.:: :.:.: : : ::..: .:.:: .:.:..::: :.
HAZARA    KVILSGKSTSIIKLEEKTGMQWSLGSESAAEEKRLLVSILDYTQVYSSTFQYITGDRTVS
             840       850       860       870       880       890

        1160      1170      1180      1190      1200      1210
CCHFV     EWPKATCTGDCPERCGCTSSTCLHKEWPHSRNWRCNPTWCWGVGTGCTCCGLDVKDLFTD
          ::::::::::::.::::..:.::.: :::::::::::::::::::::::::.:.   :.
HAZARA    EWPKATCTGDCPDRCGCSTSSCLYKSWPHSRNWRCNPTWCWGVGTGCTCCGVDILRPFNK
             900       910       920       930       940       950

        1220      1230      1240      1250      1260      1270
CCHFV     YMFVKWKVEYIKTEAIVCVELTSQERQCSLIEAGTRFNLGPVTITLSEPRNIQQKLPPEI
          :. .:: .::..:...::::::.:::.:...:::..: .::: ...:.:.:.: ::: ::
HAZARA    YFVTKWTTEYVRTDVLVCVELTDQERHCDVVEAGSQFVIGPVRVVVSDPQNVQTKLPSEI
             960       970       980       990      1000      1010

        1280      1290      1300      1310      1320      1330
CCHFV     ITLHPRIEEGFFDLMHVQKVLSASTVCKLQSCTHGVPGDLQVYHIGNLLKGDKVNGHLIH
          .:..    .   :.::. ...::...::::::::: :::.:. :  ::.. .. .:  .
HAZARA    LTIQKLEGNQVVDIMHATSIVSAKNACKLQSCTHGSPGDMQILHTDNLIQHSHDGGLNLA
            1020      1030      1040      1050      1060      1070

        1340      1350      1360      1370      1380      1390
CCHFV     KIEPHFNTSWMSWDGCDLDYYCNMGDWPSCTYTGVTQHNHASFVNLLNIETDYTKNFHFH
```

```
         ..:  :..::::.::::::::. :.::::::::....:  :: :::: :..  . ::::
HAZARA DLNPLVNSTWMSWEGCDLDYYCTTGSWPSCTYTGINSENTESFDNLLNTESNLCERFHFH
         1080      1090      1100      1110      1120      1130

     1400      1410      1420      1430      1440      1450
CCHFV  SKRVTAHGDTPQLDLKARPTYGAGEITVLVEVADMELHTKKIEISGLKFASLACTGCYAC
       :::..: :.: :.:::.::. :.::..:::.:  .:::.::: ..::.: .:.:.:::::
HAZARA SKRISASGSTLQMDLKGRPNSGGGELSVLVDVKGLELHSKKISLKGLSFKTLSCSGCYAC
         1140      1150      1160      1170      1180      1190

     1460      1470      1480      1490      1500      1510
CCHFV  SSGISCKVRIHVDEPDELTVHVKSDDPDVVAASSSLMARKLEFGTDSTFKAFSAMPKTSL
       :::.:: :......:::.:::..: .::...: .:..::..  :  :  ..::..    ..
HAZARA SSGLSCTVEVRIERPDEFTVHLRSVSPDIAVAEGSIIARRMTGGPLSRLRAFAVRKVKKI
         1200      1210      1220      1230      1240      1250

     1520      1530      1540      1550      1560      1570
CCHFV  CFYIVEREHCKSCSEEDTKKCVNTKLEQPQSILIEHKGTIIGKQNSTCTAKASCWLESVK
       :: :::. .::.:..::: ::....:. :..::.::::::: .:: ::..  .:: ::..
HAZARA CFEIVEKSYCKDCKNEDTTKCIEVELQPPKDILLEHKGTIIKRQNETCVSGLQCWTESAS
         1260      1270      1280      1290      1300      1310

     1580      1590      1600      1610         1620      1630
CCHFV  SFFYGLKNMLSGIFGNVFMGIFLFLAPFILLILFFMFG---WRILFCFKCCRRTRGLFKY
       ::  :. ... . .:.. .:: : : :  ...::: .:   ...  ::.::   ::: .
HAZARA SFVSGVGSFFRNYLGSITLGIVLTLLPVAVVLLFFCYGDKLFKLCSCFRCC---RGLSRG
         1320      1330      1340      1350      1360         1370

        1640      1650      1660      1670      1680
CCHFV  RHLKDDEETGYRRIIEKLNNKKGKNKLLDGERLADRRIAELFSTKTHIG
       .  :. .:   :  ..:.. :.:.  :.  :.   : :: :.: :
HAZARA KVRKELDEDELRNKLKKFS-KEGE--LFGKEKKDARTIALLLSGKGKNYKELV
            1380      1390         1400      1410      1420
```

CCHFV, Crimean-Congo haemorrhagic fever virus; HAZV, Hazara virus.

Supplementary Figure S3. Example of MS/MS spectrum (from Mascot) of peptide NDGPGDHITFCNGSVVTK (m/z 640.291500, 3+) with deamidation on N346 confirming that this site is occupied in HAZV.

Gn.MS/MS Fragmentation of **NDGPGDHITFCNGSVVTK**
Found in **gi|111154390** in **NCBInr**, glycoprotein precursor [Hazara virus]

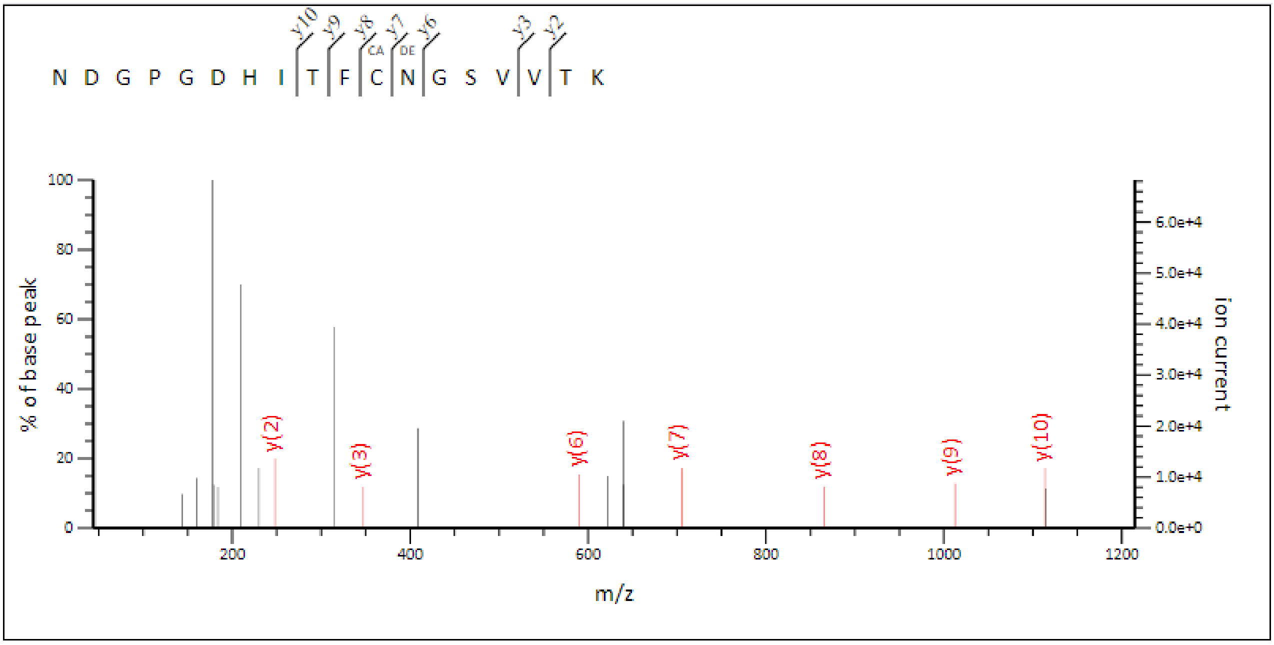

Monoisotopic mass of neutral peptide Mr(calc): 1917.8527
Variable modifications:
C11 : Carbamidomethyl (C)
N12 : Deamidated (N) **Ions Score:** 39 **Expect:** 0.00021
Matches : 7/252 fragment ions using 13 most intense peaks

Score	Mr(calc)	Delta	Sequence
39.2	1917.8527	-0.0001	NDGPGDHITFCNGSVVTK

HAZV, Hazara virus; MS, mass spectrometry.

Supplementary Figure S4. Uninfected and HAZV-infected SW13 cells are susceptible to iminosugar-mediated ER α-glucosidase inhibition. SW13 cells (assayed in technical duplicate) were left untreated or treated with 100 μM 2THO-DNJ, *N*N-DNJ, *N*N-DGJ or ribavirin, or 316 μM *N*B-DNJ or *N*B-DGJ for 3 days (**A**, **B**) or 6 days (**C**, **D**). In (**A**, **B**), cells were either mock- or HAZV-infected on day 0, immediately prior to drug treatment. In (**C**, **D**), cells were drug-treated for 3 days prior to mock- or HAZV-infection, after which the same drug treatment was continued for 3 days. At the end of the culture period, cells were lysed and FOS species purified and detected by NP-HPLC.

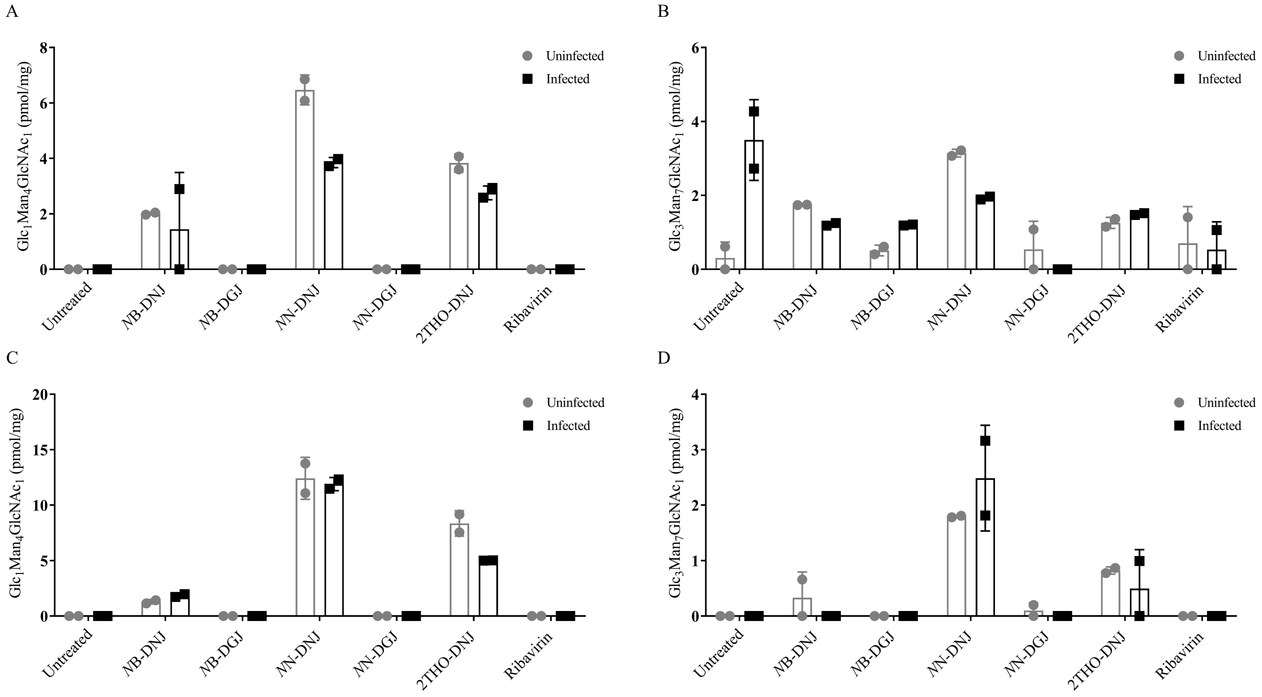

2THO-DNJ, *N*-8′-(2″-tetrahydrofuranyl)-octyl-deoxynojirimycin; ER, endoplasmic reticulum; FOS, free oligosaccharide; HAZV, Hazara virus; *N*B-DGJ, *N*-butyl-deoxygalactojirimycin; *N*B-DNJ, *N*-butyl-deoxynojirimycin; *N*N-DGJ, *N*N-deoxygalactonojirimycin; *N*N-DNJ, *N*-nonyl-deoxynojirimycin; NP-HPLC, normal phase-high performance liquid chromatography.

Supplementary Figure S5. Cytotoxicity of iminosugars and ribavirin in SW13 (**A**) and Vero cells (**B**). SW13 and Vero cells (assayed in technical quadruplicate) were treated with drug as indicated for 3 days prior to cell viability assessment by MTS assay. Data were normalised to untreated and represented as mean ± standard deviation.

A B

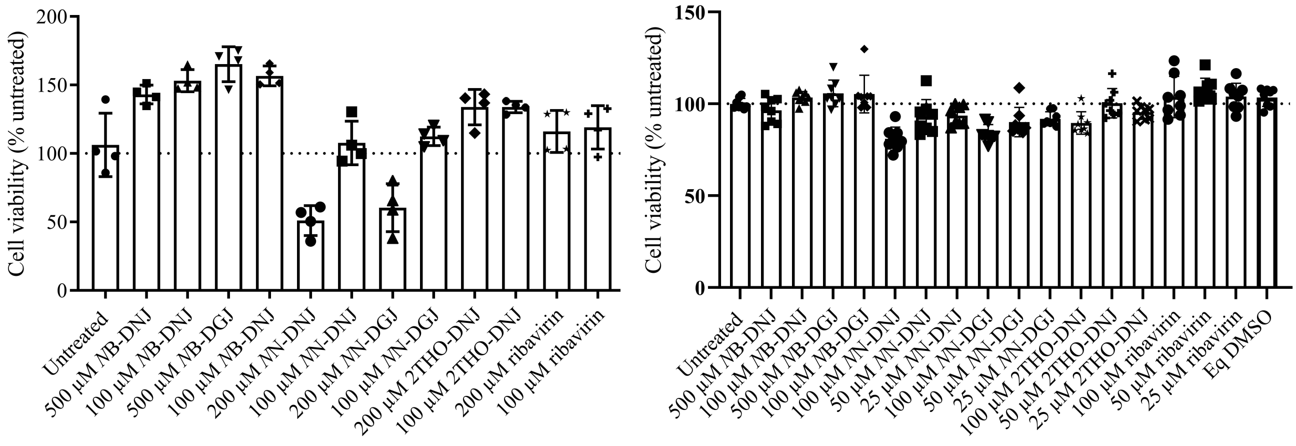

2THO-DNJ, *N*-8′-(2″-tetrahydrofuranyl)-octyl-deoxynojirimycin; MTS, 3-(4,5-dimethyl-2-yl)-5-(3-carboxymethoxyphenyl)-2-(4-sulfophenyl)-2H-tetrazolium; *N*B-DGJ, *N*-butyl-deoxygalactojirimycin; *N*B-DNJ, *N*-butyl-deoxynojirimycin; *N*N-DGJ, *N*N-deoxygalactonojirimycin; *N*N-DNJ, *N*-nonyl-deoxynojirimycin.

Supplementary Figure S6. Uninfected Vero cells are susceptible to iminosugar-mediated ER α-glucosidase inhibition. Vero cells (assayed in technical triplicate) were left untreated or treated with 316 μM *N*B-DNJ, *N*B-DGJ or 100 μM ribavirin for 3 days. At the end of the culture period, cells were lysed and FOS species purified and measured by NP-HPLC. (**A**) $Glc_1Man_4GlcNAc_1$ and (**B**) $Glc_3Man_7GlcNAc_2$ FOS species were detected: these are diagnostic for ER α-glucosidase II and I inhibition, respectively. These were normalised to total protein content and plotted as mean ± standard deviation.

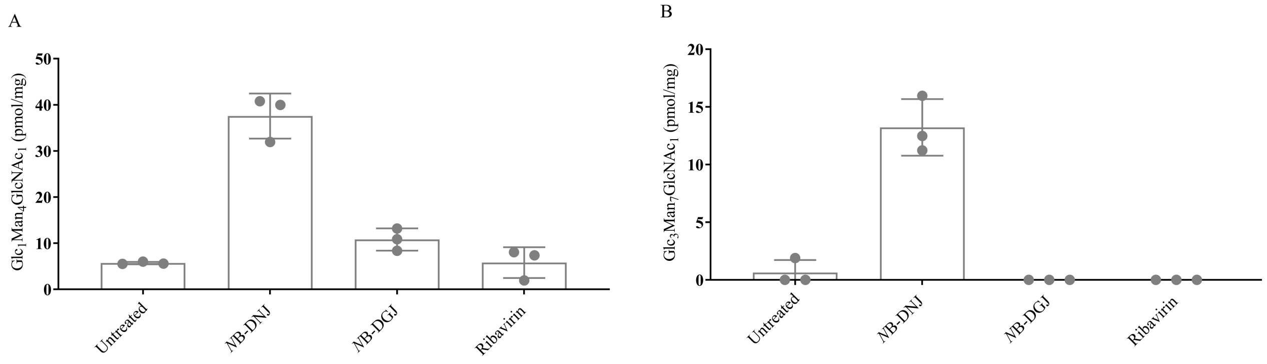

ER, endoplasmic reticulum; FOS, free oligosaccharide; *N*B-DGJ, *N*-butyl-deoxygalactojirimycin; *N*B-DNJ, *N*-butyl-deoxynojirimycin; NP-HPLC, normal phase-high performance liquid chromatography.
